# The role of multimorbidity in suspected dementia among elderly in Shanghai, China: a community-based cross-sectional study

**DOI:** 10.3389/fpsyt.2025.1596281

**Published:** 2025-07-17

**Authors:** Tingting Zhu, Changying Wang, Yunwei Zhang, Xiaqing Wang, Yuhong Niu

**Affiliations:** Shanghai Health Development Research Center (Shanghai Medical Information Center), Research Management Affairs Department, Shanghai, China

**Keywords:** elderly, dementia, multimorbidity, diabetes, hyperlipidemia

## Abstract

**Introduction:**

Many studies have shown some chronic diseases are one of the key risk factors for accelerating cognitive decline. and multimorbidity are common in the elderly population. The evidence of the impact of multimorbidity on dementia among elderly people in China is scarce in detail. This study was performed to examine the association between the prevalence of suspected dementia and multimorbidity, as well as pattern of multimorbidity among the elderly in Shanghai.

**Methods:**

This was a cross-sectional study, with 5040 elderly individuals from 21 communities enrolled. The prevalence of suspected dementia was assessed using the Mini-Mental State Examination (MMSE). In addition, the diagnosed chronic diseases including hypertension, diabetes, hyperlipidemia and coronary heart disease (CHD) were investigated such that multimorbidity was defined as individuals suffering from two or more chronic diseases at the same time. Binary logistic regression models were utilized to analyze the impact of multimorbidity and its patterns on suspected dementia.

**Results:**

Data of 4945 older adults were analyzed. The overall prevalence of suspected dementia and multimorbidity were 15.73% and 35.98%. The influencing factors of dementia from the perspective of single disease, including diabetes, hyperlipidemia, abnormal control of blood glucose and abnormal control of blood lipid. Multivariate analysis showed multimorbidity (OR=1.491, 95%*CI*: 1.260-1.765) was significantly negatively associated with dementia, and the risk of dementia in elderly individuals with 2, 3 or more chronic diseases was 1.283 (95%*CI*: 1.058-1.555) and 2.034 (95%*CI*: 1.600-2.586) times greater, respectively, than those who with no multimorbidity. Notably, elderly individuals with both diabetes and hyperlipidemia had the highest risk of dementia (OR=3.253, 95%*CI*: 1.705-6.207).

**Conclusion:**

Multimorbidity played a negative role in dementia among elderly people, dementia risk increases with the number of comorbidities, and the combination of diabetes and hyperlipidemia accentuates dementia risk at a greater level.

## Introduction

With the increasing prevalence of dementia, dementia and its associated care burden have become important public health issues. The number of dementia patients worldwide will increase from 57.4 million in 2019 to 150 million in 2050, with 25% of patients in China having dementia (1, 2). According to a nationally representative cross-sectional study in 2018, 7.1% of adults aged 60–108 in China suffer from dementia (3). By 2022 the number of dementia patients aged 60 years and older is expected to increase to 15 million, and dementia has become the fifth leading cause of death for both urban and rural residents (4, 5). At present, there are no drugs can effectively cure or slow the progression of cognitive decline (6), so identifying relevant risk factors is key to preventing dementia. Environmental factors play a greater role in dementia (6), in which chronic diseases such as hypertension, diabetes and cerebrovascular diseases have been demonstrated to be individually associated with dementia in several previous studies (7–11).

Given the decline in physical function, the elderly have become a high-risk group of chronic diseases, and coupled with the increase in life expectancy, multimorbidity which is often defined as the presence of two or more chronic diseases in an individual at the same time has become increasingly common with ageing populations (12). According to a 2023 survey, the prevalence of multimorbidity was 24.5% in high-income countries, 33.9% in upper-middle-income countries, and 8.1% in lower-middle-income countries (13). In China, more than 30% of people aged 60 and above suffer from multimorbidity, and the proportion of multimorbidity is increasing with age (14, 15). Multimorbidity has a negative impact on physical health and cognitive function, as well as on quality of life (16, 17). As mentioned above, multimorbidity and dementia associated with aging have become common health challenges that must be addressed in the future. According to data published by the World Health Organization (WHO), the global population aged 60 and over is expected to grow to 2.1 billion by 2050, accounting for 22% of the world’s population. China is currently in an accelerated period of population aging, and the elderly population aged 60 and above will exceed 400 million by approximately 2035, accounting for more than 30% of the total population, and will enter the stage of heavy aging. As the elderly population grows, cognitive decline and comorbidities of various chronic diseases pose significant challenges to the public health system.

To this end, a report by the Lancet Commission on Dementia Prevention, Intervention, and Care emphasized the necessity of considering multimorbidity in relation to dementia in 2020 (6). Many studies have shown that chronic conditions such as stroke and other cerebrovascular diseases are associated with faster cognitive decline and a greater risk of dementia progression (18–20). In addition, individuals with dementia are more susceptible to other chronic illnesses than the general elderly population is (21, 22). However, most available studies focused on individual condition, so the knowledge gap is limited studies focusing on multimorbidity and dementia risk. Here, this study aimed to investigate the prevalence of multimorbidity and suspected dementia and quantify the impact of multimorbidity on suspected dementia among community-dwelling elderly individuals aged 60 years and older in Shanghai to provide a reference for research on the prevention of dementia and multimorbidity.

## Materials and methods

### Study design

This was a cross-sectional study conducted in Shanghai by the Shanghai Health and Development Research Center (Shanghai Institute of Medical Science and Technology Information) in August 2021. The research project was approved by the Ethics Committee of the Shanghai Health Development Research Center (Approval Number 2020001). All participants signed voluntarily the written informed consent form prior to commencing the study.

### Sample size calculation

The minimum sample size for this study was calculated using the following formula for cross-sectional studies:


$$\text{N}=\frac{\mu^{2}\alpha_{/2}\pi \;(1-\pi )}{\delta^{2}}$$

where μ_α/2_ = 1.96 when α = 0.05, π is the prevalence of dementia [which was 7.4% according to a previous study (23)], and δ is the admissible error (which was 0.1π here). According to the formula, the estimated sample size was approximately 4,808.

### Study population and procedure

A multistage random sampling method was utilized to select a representative sample as follows. In the first stage, two districts of the seven central urban areas (Xuhui District and Putuo District) and one of the eight rural areas (Qingpu District) were randomly selected based on economic level and proportion of permanent residents. In the second stage, seven communities each from the three sampled districts were randomly selected using a random number table. In the third stage, all people over 60 years old in the 21 selected communities were numbered, 240 people who met the inclusion and exclusion criteria were interviewed in each community.

The elderly population in the selected community formed the sampling frame, and participants aged 60 years and older who voluntarily participated in this study were included in our study. However, participants were excluded if they had severe hearing impairment or a language barrier. The theoretical sample size was 5040 people, of which 89 were not investigated for various reasons (response rate: 98.23%). Of the remaining 4951 elderly adults, 6 were excluded due to incomplete data. Ultimately, a total of 4,945 elderly adults were included in the data analysis in this study.

### Measurements

We collected the main general information through questionnaire, including demographic characteristics and lifestyle behaviors. Demographic information, including gender, age, education level, and occupation, was collected. Education level was divided into 4 groups according to the level of education of the study population: illiterate (<1 year), primary school (1–6 years), middle school (7–12 years), and college (13 years and above). Occupation was dichotomized into mental labor and manual labor according to the nature of the work that the study population had been engaged in before retirement or all along. Lifestyle behaviors, including living situation, smoking, alcohol and tea consumption, and exercise. Living situation includes residence location, which is divided into urban and rural areas, and living arrangement, which is divided into solitary and non-solitary living.

The Mini-Mental State Examination (MMSE) was used to older adults as a screening tool for suspected dementia, which is the most widely used cognitive examination scale and has been proven to have good reliability and validity in the Chinese population with a Cronbach’s α of 0.893 (24, 25). The scale consists of 30 items covering 5 dimensions, including time orientation, memory, attention and calculation, recall ability, and language ability, with each entry scoring 1 point for a correct answer, 0 points for an incorrect or unknown answer, and a total score of 0–30 points, with higher scores indicating better cognitive function. The threshold of suspected dementia was determined based on the education level, with an assessment score of ≤17 points for the illiterate group, ≤20 points for the primary school group, and ≤24 points for the junior high school and above group in this study (24).

We investigated the older adults on the common chronic diseases, including hypertension, diabetes, hyperlipidemia and coronary heart disease (CHD), which have the highest incidence rates among elderly people in Shanghai. The participants were asked questions about disease onset and disease indicator control. Normal blood pressure control was defined as a systolic blood pressure<140 mmHg and/or diastolic blood pressure<90 mmHg. Normal blood glucose control was defined as fasting plasma glucose<7.0 mmol/L. Normal blood lipid control was defined as low-density lipoprotein cholesterol<3.4 mmol/L. The chronic disease information self-reported by research participants must be diagnosed by clinical doctors and validated through health records from community health service centers. Elderly patients with multimorbidity were defined as those who suffered from two or more chronic diseases at the same time and were divided into 11 multimorbidity pattern groups according to the combination of different diseases.

### Data collection

A data survey was conducted with the support of community health service centers, and all the data were obtained through face-to-face interviews with trained community doctors who spent 20 to 30 minutes with each respondent. For quality control, all investigators received cognitive function assessment training to ensure mastery of the scale content and assessment methods. Additionally, telephone calls were made to more than 20% of the respondents who were randomly selected to ensure the authenticity of the survey data. The data were double-checked to remove participants who had obvious logical errors and incomplete information, and data entry into Epidata 3.1 was performed by two people separately.

### Statistical analysis

The statistical analysis were performed using SPSS V.29.0 software. Categorical data were summarized as frequency counts and percentages, whereas continuous data were described as the mean ± SD. The distributions of categorical variables and rank variables were analyzed using the Pearson chi-square test and the rank-sum test, respectively. Binary logistic regression was used to identify the impact of chronic diseases and multimorbidity on dementia using both univariate (crude) and multivariable (adjusted) models. The strength of association was estimated as odds ratios (OR) and 95% confidence intervals (*CI*). All the statistical tests were two-tailed, and *p*<0.05 was considered to indicate statistical significance.

## Results

### Characteristics of the study sample

A total of 4945 community-dwelling elderly people aged 60 years and above with a mean age of 71.45 ± 7.27 years were included in this study; 3245 (65.62%) lived in urban areas, 2219 (44.87%) were male. Hypertension was the most common disease, with a prevalence as high as 60.67%, and more than half of the patients had blood pressure controlled within the normal range. All the characteristics except smoking were significantly different between the dementia group and the non-dementia group, as shown in **Table 1**.

**Table 1:** Characteristics of the elderly people in the communities (*n*=4945).

Characteristics	Total (*n*=4945)	Dementia group (*n*=778)	Non dementia group (*n*=4167)	χ2/Z value	*P*
Gender				4.206	0.041
Male	2219 (44.87)	323 (41.52)	1896 (45.50)		
Female	2726 (55.13)	455 (58.48)	2271 (54.50)		
Age, years				-13.771	<0.001
60-64	860 (17.39)	88 (11.31)	772 (18.53)		
65-69	1406 (28.43)	148 (19.02)	1258 (30.19)		
70-74	1221 (24.69)	145 (18.64)	1076 (25.82)		
75-79	718 (14.52)	121 (15.55)	597 (14.33)		
≥80	740 (14.96)	276 (35.48)	464 (11.14)		
Education level, years				-10.024	<0.001
<1	389 (7.87)	146 (18.77)	243 (5.83)		
1-6	1059 (21.42)	178 (22.88)	881 (21.14)		
7-12	2817 (56.97)	393 (50.51)	2424 (58.17)		
≥13	680 (13.75)	61 (7.84)	619 (14.85)		
Occupation				76.286	<0.001
Manual labor	2884 (58.32)	564 (72.49)	2320 (55.68)		
Mental labor	2061 (41.68)	214 (27.51)	1847 (44.32)		
Residence location				41.705	<0.001
Urban areas	3245 (65.62)	432 (55.53)	2813 (67.51)		
Rural areas	1700 (34.38)	346 (44.47)	1354 (32.49)		
Living arrangement				26.294	<0.001
Solitary living	530 (10.72)	124 (15.94)	406 (9.74)		
Non-solitary living	4415 (89.28)	654 (84.06)	3761 (90.26)		
Smoking	745 (15.07)	100 (12.85)	645 (15.48)	3.531	0.063
Alcohol drinking	996 (20.14)	128 (16.45)	868 (20.83)	7.812	0.005
Tea consumption	2356 (47.64)	336 (43.19)	2020 (48.48)	7.351	0.007
Exercise	4025 (81.40)	531 (68.25)	3494 (83.85)	105.322	<0.001
Hypertension	3000 (60.67)	521 (66.97)	2479 (59.49)	15.353	<0.001
Diabetes	1215 (24.57)	233 (29.95)	982 (23.57)	14.41	<0.001
Hyperlipidemia	884 (17.88)	179 (23.01)	705 (16.92)	16.557	<0.001
CHD	1005 (20.32)	193 (24.81)	812 (19.49)	11.462	0.001
Blood pressure control				15.353	<0.001
Normal	2540 (51.37)	441 (56.68)	2099 (50.37)		
Abnormal	460 (9.30)	80 (10.28)	380 (9.12)		
Blood glucose control				17.028	<0.001
Normal	963 (19.47)	193 (24.81)	770 (18.48)		
Abnormal	252 (5.10)	40 (5.14)	212 (5.09)		
Blood lipid control				50.802	<0.001
Normal	692 (13.99)	114 (14.65)	578 (13.87)		
Abnormal	192 (3.88)	65 (8.35)	127 (3.05)		

The χ2 test was used to evaluate categorical variables, and the Z test was used to evaluate rank variables. CHD, coronary heart disease.

### Prevalence of suspected dementia and multimorbidity

A total of 778 (15.73%) older adults were assessed as suspected dementia by MMSE, 351 of whom had multimorbidity, with the prevalence of suspected dementia was 19.7% (351/1779) in the multimorbidity group. There were 1,779 (35.98%) elderly individuals with multimorbidity, one quarter of them suffered from two diseases approximately. Univariate analysis showed that an increased incidence of suspected dementia was associated with more number of diseases. The prevalence of suspected dementia in the older adults with 2 diseases, 3 or more diseases were 17.71% (217/1225), 24.19% (134/554). Besides, we found significant associations between multimorbiditiy patterns and suspected dementia. Elderly with diabetes and CHD had the highest suspected dementia prevalence, as shown in **Table 2**.

**Table 2:** Multimorbidity and suspected dementia (*n*=4945).

Variables	Total (N=4945)	Suscepted dementia group (N=778)	Non dementia group (N=4167)	χ2/Z value	*P*
Multimorbidity	1779 (35.98)	351 (45.12)	1428 (34.27)	33.485	<0.001
Number of multimorbidity				45.543	<0.001
2	1225 (24.77)	217 (27.89)	1008 (24.19)		
≥3	554 (11.20)	134 (17.22)	420 (10.08)		
Multimorbiditiy patterns				65.098	<0.001
Hypertension + diabetes	517 (10.46)	430 (55.27)	87 (2.09)		
Hypertension+ CHD	330 (6.67)	266 (34.19)	64 (1.54)		
Hypertension+ hyperlipidemia	221 (4.47)	180 (23.14)	41 (0.98)		
Hyperlipidemia + CHD	61 (1.23)	54 (6.94)	7 (0.17)		
Diabetes + hyperlipidemia	49 (0.99)	34 (4.37)	15 (0.36)		
Diabetes+CHD	47 (0.95)	44 (5.66)	3 (0.07)		
Hypertension + hyperlipidemia + CHD	170 (3.44)	132 (16.97)	38 (0.91)		
Hypertension + diabetes + hyperlipidemia	130 (2.63)	94 (12.08)	36 (0.86)		
Hypertension + diabetes + CHD	128 (2.59)	92 (11.83)	36 (0.86)		
Diabetes + hyperlipidemia + CHD	20 (0.40)	17 (2.19)	3 (0.07)		
Hypertension + diabetes + hyperlipidemia + CHD	106 (2.14)	85 (10.93)	21 (0.50)		

The χ2 test was used to evaluate categorical variables, and the Z test was used to evaluate rank variables. CHD, coronary heart disease.

### Effect of chronic diseases on suspected dementia

Based on the results of the binary logistic regression models, the differences in chronic diseases between the suspected dementia group and the non-dementia group were examined. In the crude model, hypertension, diabetes, hyperlipidemia, CHD, normal and abnormal blood pressure control, abnormal blood glucose control and abnormal blood lipid control had significant impacts on suspected dementia. After adjusting for variables with statistical significance other than disease variables in **Table 1**, the results indicated that diabetes, hyperlipidemia, abnormal control of blood glucose and abnormal blood lipid levels had negative effects on suspected dementia, as shown in **Table 3**.

**Table 3:** Associations between chronic diseases and suspected dementia (*n*=4945).

Variables	Crude model			Adjusted model		
	OR	95%*CI*	*P*	OR	95%*CI*	*P*
Hypertension	1.380	1.174-1.623	<0.001	1.001	0.839-1.195	0.989
Diabetes	1.387	1.171-1.642	<0.001	1.384	1.151-1.663	0.001
Hyperlipidemia	1.467	1.219-1.767	<0.001	1.583	1.284-1.952	<0.001
CHD	1.363	1.139-1.632	0.001	1.073	0.875-1.316	0.497
Blood pressure control						
Normal	1.380	1.168-1.630	<0.001	1.024	0.853-1.229	0.798
Abnormal	1.383	1.051-1.820	0.021	1.049	0.766-1.437	0.763
Blood glucose control						
Normal	1.103	0.777-1.565	0.584	0.973	0.654-1.447	0.893
Abnormal	1.465	1.221-1.757	<0.001	1.537	1.258-1.877	<0.001
Blood lipid control						
Normal	1.140	0.916-1.419	0.241	1.190	0.934-1.516	0.16
Abnormal	2.958	2.167-4.038	<0.001	3.444	2.455-4.831	<0.001

Adjusted for gender, age, education, occupation, residence location, living arrangement, alcohol drinking, tea consumption, and exercise. CHD, coronary heart disease.

### Role of multimorbidity in suscepted dementia

Additionally, binary logistic regression analysis of the effect of multimorbidity on suspected dementia showed that participants with multimorbidity had an increased risk of dementia in the crude model. This relationship remained significant after adjusting for other covariates. Furthermore, participants were divided into 3 groups according to the number of multimorbidity. The analysis revealed that there was a statistically significant difference between groups with different numbers of multimorbidities and those without multimorbidities in the crude model. Similar results were observed in the adjusted model. In other words, participants with 2, 3 or more chronic diseases were 1.283 (95%*CI*: 1.058-1.555) times and 2.034 (95%*CI*: 1.600-2.586) times more likely to have dementia than those with 0 or 1 chronic disease, respectively. For the multimorbidity patterns, negative associations were found between the 8 combinations of multimorbidity and suspected dementia in the crude model. However, after controlling for other confounding factors, it was observed that significant associations between the combinations of diabetes and hyperlipidemia in the binary multimorbidity groups and suspected dementia, and all combinations except diabetes, hyperlipidemia and CHD in the ternary multimorbidity groups. The strongest association was for the combination of diabetes and hyperlipidemia, with an odds ratio of 3.253 (95%*CI*: 1.705-6.207), which means that elderly with both diabetes and hyperlipidemia were 3.253 times more likely to had suspected dementia than those with 0 or 1 chronic condition, as shown in **Table 4**.

**Table 4:** Associations between multimorbidity and suspected dementia (*n*=4945).

Variables	Crude model			Adjusted model		
	OR	95%*CI*	*P*	OR	95%*CI*	*P*
Multimorbidity (Yes)	1.577	1.35-1.841	<0.001	1.491	1.260-1.765	<0.001
Number of multimorbidity						
2	1.381	1.155-1.651	<0.001	1.283	1.058-1.555	0.011
≥3	2.047	1.643-2.549	<0.001	2.034	1.600-2.586	<0.001
Multimorbiditiy combinations						
Hypertension +diabetes	1.298	1.009-1.670	0.043	1.254	0.959-1.639	0.098
Hypertension+CHD	1.543	1.153-2.065	0.004	1.149	0.835-1.581	0.394
Hypertension+hyperlipidemia	1.461	1.025-2.082	0.036	1.439	0.982-2.107	0.062
Hyperlipidemia + CHD	0.832	0.376-1.839	0.649	1.117	0.486-2.566	0.794
Diabetes +hyperlipidemia	2.830	1.528-5.240	0.001	3.253	1.705-6.207	<0.001
Diabetes+CHD	0.437	0.135-1.415	0.167	0.500	0.151-1.655	0.256
Hypertension + hyperlipidemia + CHD	1.847	1.269-2.687	0.001	1.804	1.204-2.702	0.004
Hypertension + diabetes +hyperlipidemia	2.457	1.651-3.656	<0.001	2.894	1.909-4.387	<0.001
Hypertension + diabetes +CHD	2.510	1.685-3.739	<0.001	2.123	1.361-3.311	0.001
Diabetes + hyperlipidemia +CHD	1.132	0.33-3.879	0.844	1.205	0.339-4.278	0.773
Hypertension + diabetes + hyperlipidemia + CHD	1.585	0.972-2.583	0.065	1.544	0.913-2.612	0.105

Adjusted for gender, age, education, occupation, residence location, living arrangement, alcohol drinking, tea consumption, and exercise. CHD, coronary heart disease.

## Discussion

With the aging population and increasing life expectancy, dementia and chronic diseases have become common. The burden of multimorbidity and the care it brings have gradually become a public health problem (29). Studies have shown that multimorbidities are common in patients with dementia (26, 27). Investigating the prevalence of dementia among elderly people in the community and exploring the impact of chronic diseases and multimorbidity on dementia is highly important for the precise prevention and control of dementia.

This study assessed the cognitive function of community-dwelling older adults aged 60 years and older in Shanghai, China, by the MMSE. Based on a large population, the prevalence of suspected dementia was 15.73%, which was higher than that reported by Song, Y H et al., who reported that 7.62% of elderly residents had dementia in Xiamen, China (28), and higher than the overall prevalence of dementia in China reported by Jia, L F et al., 2020, which was 6.0% (30). An explanation for the variance in these estimates may be the differences among the included subjects, who were more than half were aged 70 years and above, and advanced age has been shown to be an immutable risk factor for dementia (31, 32); therefore, the prevalence of suspected dementia was relatively high in this study. Moreover, this variance may also be related to factors such as inconsistencies in the screening tools, survey methods and diagnostic criteria used in different surveys. In this study, only the MMSE was used for screening, and there is a possibility of overestimation in the evaluation results, but it still shows that the cognitive function status of elderly people aged 60 and above in Shanghai is poor.

With respect to the association between chronic conditions and suspected dementia, this study revealed that elderly individuals with diabetes or abnormal blood sugar after control were more likely to have suspected dementia. This finding was similar to the analysis results of data from nearly 458,000 diabetes patients aged 50 years and over who were followed up for 6 years, which indicated that higher or unstable glycosylated hemoglobin levels were associated with an increased risk of suspected dementia (33). This may be because hyperglycemic states contribute to vasculopathy, inflammatory responses, and oxidative stress, causing damage to neurons and glial cells in the brain, which prevents them from transmitting or receiving signals properly, leading to cognitive decline (33). In addition, we found a negative association between hyperlipidemia or abnormal control of hyperlipidemia and suspected dementia, and older adults with abnormal control of hyperlipidemia had a 3.444 times higher risk of suspected dementia compared to those without hyperlipidemia. There is a lack of consistency in the results of previous studies on the relationship between lipid levels and the risk of developing dementia (34). However, most studies support the idea that an increase in total cholesterol or low-density cholesterol (LDL-C) increases the risk of suspected dementia, which may be related to the role of cholesterol in the regulation of *β*-amyloid production (35).

Furthermore, after stratifying the subjects based on the number of diseases, it was found that the multimorbidity group had higher suspected dementia prevalence, suggesting that older adults with multiple chronic diseases were more likely to develop suspected dementia, which is consistent with other studies conducted in China and in other countries (36, 37). A more detailed investigation of the association between multimorbidity and suspected dementia revealed that multimorbidity still has a significant impact on cognitive decline in elderly people, and the greater the number of multimorbidities is, the greater the risk of suspected dementia. A previous study based on a large-scale prospective cohort also found the similar dose–response relationship between multimorbidity and dementia risk (38). With respect to the specific compositions of multimorbidity, the most prevalent multimorbidity pattern was hypertension and diabetes among all participants and the dementia group, and the combination of diabetes and hyperlipidemia increased the risk of suspected dementia to the greatest extent among the 4 multimorbidity combinations that had significant negative effects on suspected dementia in the elderly population. This finding is consistent with a previous study in which patients with diabetes and hyperlipidemia had an elevated risk of dementia (39). One explanation is that some common pathogenetic mechanisms could be involved in the relationship between multimorbidity as a whole and dementia, which exacerbates cognitive decline compared to the independent association of chronic conditions (42). One alternative explanation might be that multiple morbidities can lead to polypharmacy, and drug interactions may have a negative impact on cognitive abilities, which in turn increases the risk of dementia (40, 41).

Multimorbidity are common in the elderly population and cause obvious damage to cognitive function. However, there are few studies on the association between multimorbidity and dementia, and public attention is insufficient. Given the findings of this study, it is time to draw the attention of the government and relevant departments to the management of chronic diseases and dementia among elderly people. First, increased investment in the management of chronic diseases, including establishing resident health records and setting up specialized outpatient clinics for multimorbidity, is needed. Second, community doctors need to conduct chronic disease surveys and regular follow-up on elderly people, achieve continuous and dynamic monitoring, and carry out targeted preventive interventions for patients to reduce the occurrence of adverse outcomes. Moreover, health education is crucial for individuals. Science popularization activities should be strengthened to raise awareness of health management in the elderly population, regularly monitor blood glucose and lipid levels, and take the initiative to comply with doctors’ instructions to keep the indicators within the normal or reasonable range. In addition, multimorbidities should be included in the assessment indicators of cognitive decline, and the screening of cognitive disorders for elderly people in the community should be carried out to promote early detection of and intervention in the state of cognitive decline and to pay attention to multimorbidities to strengthen the prevention of related risk factors. This is to slow the decline in cognitive function and reduce the risk of dementia.

The strengths of this study lay in the community population sampling and attention to the impact of specific multimorbidity patterns on dementia. Several limitations should be acknowledged. The causal relationship between chronic diseases and dementia could not be determined due to the cross-sectional study design. Recall bias might exist because of disease history and control status relied on self-reported. Our study only measured the impact of multimorbidities of four chronic diseases on suspected dementia, and important influencing factors, such as depressive symptoms, nutritional status and sensory impairments, were not adjusted for in this study and may have confounded the results. Future studies should also investigate the prevalence of other chronic diseases and explore the associations between dementia and different multimorbidity patterns comprehensively.

## Conclusions

In summary, elderly people aged 60 years and above in Shanghai had a high prevalence of suspected dementia. The risk of suspected dementia was significantly affected by multimorbidity and increased with the number of multimorbidities, and the combination of diabetes and hyperlipidemia was the pattern with the highest risk of suspected dementia. Therefore, when formulating dementia prevention and treatment measures, comprehensive consideration should be given to the prevalence of chronic diseases and the control of disease indicators in elderly people, and timely intervention and treatment should be carried out to minimize the occurrence of multimorbidities. At the same time, we should focus on the multimorbidity group and implement cognitive function screening and risk factor prevention and control to promote early detection of cognitive decline and early intervention to reduce the incidence of dementia.

## Data Availability

The original contributions presented in the study are included in the article/supplementary material. Further inquiries can be directed to the corresponding authors.

## References

[1] Nichols E Steinmetz JD Vollset SE Fukutaki K Chalek J Abd-Allah F. Estimation of the global prevalence of dementia in 2019 and forecasted prevalence in 2050: An analysis for the Global Burden of Disease Study 2019. Lancet Public Health. (2022) 7:E105–25. doi: 10.1016/S2468-2667(21)00249-8, PMID: 34998485 PMC8810394

[2] Jia LF Quan MN Fu Y Zhao T Li Y Wei CB. Dementia in China: Epidemiology, clinical management, and research advances. Lancet Neurol. (2020) 19:81–92. doi: 10.1016/S1474-4422(19)30290-X, PMID: 31494009

[3] Chen H Huang Y Lv X Xu X Ma Y Wang H. Prevalence of dementia and the attributable contributions of modifiable risk factors in China. Gen Psychiatr. (2023) 36:e101044. doi: 10.1136/gpsych-2023-101044, PMID: 37565235 PMC10410793

[4] Huang YX Li XD Liu ZF Huo JH Guo JW Chen YY. Projections of the economic burden of care for individuals with dementia in mainland China from 2010 to 2050. PloS One. (2022) 17(2):e0263077. doi: 10.1371/journal.pone.0263077, PMID: 35113895 PMC8812891

[5] Ren R Qi J Lin S Liu X Yin P Wang Z. The China alzheimer report 2022. Gen Psychiatr. (2022) 35:e100751. doi: 10.1136/gpsych-2022-100751, PMID: 35372787 PMC8919463

[6] Livingston G Sommerlad A Orgeta V Costafreda SG Huntley J Ames D. Dementia prevention, intervention, and care. Lancet. (2017) 390:2673–734. doi: 10.1016/S0140-6736(17)31363-6, PMID: 28735855

[7] Streit S Poortvliet R den Elzen W Blom JW Gussekloo J. Systolic blood pressure and cognitive decline in older adults with hypertension. Ann Fam Med. (2019) 17:100–7. doi: 10.1370/afm.2367, PMID: 30858252 PMC6411391

[8] Ma CR Yin ZX Zhu PF Luo JS Shi XM Gao X. Blood cholesterol in late-life and cognitive decline: A longitudinal study of the Chinese elderly. Mol Neurodegener. (2017) 12(1):24. doi: 10.1186/s13024-017-0167-y, PMID: 28270179 PMC5341475

[9] Zhang JJ Wu ZX Tan W Liu D Cheng GR Xu L. Associations among multidomain lifestyles, chronic diseases, and dementia in older adults: A cross-sectional analysis of a cohort study. Front Aging Neurosci. (2023) 15:1200671. doi: 10.3389/fnagi.2023.1200671, PMID: 37600519 PMC10438989

[10] Horsburgh K Wardlaw JM van Agtmael T Allan SM Ashford M Bath PM. Small vessels, dementia and chronic diseases - molecular mechanisms and pathophysiology. Clin Sci (Lond). (2018) 132:851–68. doi: 10.1042/CS20171620, PMID: 29712883 PMC6700732

[11] Wardlaw JM Horsburgh K. Introductory Editorial to the Special Themed Issue Small vessels, dementia and chronic diseases molecular - mechanisms and pathophysiology. Clin Sci (Lond). (2016) 130:1875–9. doi: 10.1042/CS20160376, PMID: 27660310

[12] Tinetti ME Fried TR Boyd CM. Designing health care for the most common chronic Condition-Multimorbidity. JAMA. (2012) 307:2493–4. doi: 10.1001/jama.2012.5265, PMID: 22797447 PMC4083627

[13] Ni Y Zhou Y Kivimäki M Cai Y Carrillo-Larco RM Xu X. Socioeconomic inequalities in physical, psychological, and cognitive multimorbidity in middle-aged and older adults in 33 countries: A cross-sectional study. Lancet Healthy Longev. (2023) 4:e618–28. doi: 10.1016/S2666-7568(23)00195-2, PMID: 37924843

[14] Fan JN Sun ZJ Yu CQ Guo Y Pei P Yang L. Multimorbidity patterns and association with mortality in 0.5 million Chinese adults. Chin Med J (Engl). (2022) 135:648–57. doi: 10.1097/CM9.0000000000001985, PMID: 35191418 PMC9276333

[15] Hu XL Huang J Lv YQ Li GX Peng XX. Status of prevalence study on multimorbidity of chronic disease in China: Systematic review. Geriatr Gerontol Int. (2015) 15:1–10. doi: 10.1111/ggi.12340, PMID: 25163532

[16] Vassilaki M Aakre JA Cha RH Kremers WK St Sauver JL Mielke MM. Multimorbidity and risk of mild cognitive impairment. J Am Geriatr Soc. (2015) 63:1783–90. doi: 10.1111/jgs.13612, PMID: 26311270 PMC4607039

[17] Falvey JR Gustavson AM Price L Papazian L Stevens-Lapsley JE. Dementia, comorbidity, and physical function in the program of All-Inclusive care for the elderly. J Geriatr Phys Ther. (2019) 42:E1–6. doi: 10.1519/JPT.0000000000000131, PMID: 28437317 PMC5650947

[18] Kadambi S Abdallah M Loh KP. Multimorbidity, function, and cognition in aging. Clin Geriatr Med. (2020) 36:569–84. doi: 10.1016/j.cger.2020.06.002, PMID: 33010895 PMC8012012

[19] Dove A Marseglia A Shang Y Grande G Vetrano DL Laukka EJ. Cardiometabolic multimorbidity accelerates cognitive decline and dementia progression. Alzheimers Dement. (2023) 19:821–30. doi: 10.1002/alz.12708, PMID: 35708183

[20] Wang Z Fei L Xu Y Deng F Zhong B. Prevalence and correlates of suspected dementia in older adults receiving primary healthcare in Wuhan, China: A multicenter cross-sectional survey. Front Public Health. (2022) 10:1032118. doi: 10.3389/fpubh.2022.1032118, PMID: 36267996 PMC9577294

[21] Browne J Edwards DA Rhodes KM Brimicombe DJ Payne RA. Association of comorbidity and health service usage among patients with dementia in the UK: A population-based study. BMJ Open. (2017) 7(3):e012546. doi: 10.1136/bmjopen-2016-012546, PMID: 28279992 PMC5353300

[22] Kaczynski A Michalowsky B Eichler T Thyrian JR Wucherer D Zwingmann I. Comorbidity in dementia diseases and associated health care resources utilization and cost. J Alzheimers Dis. (2019) 68:635–46. doi: 10.3233/JAD-180896, PMID: 30856111

[23] Wang YQ Jia RX Liang JH Li J Qian S Li JY. Dementia in China (2015-2050) estimated using the 1% population sampling survey in 2015. Geriatr Gerontol Int. (2019) 19:1096–100. doi: 10.1111/ggi.13778, PMID: 31535462

[24] Cheng Q Sun HX Ye FL Wang G Ling HW Chen SD. Dementia among elderly in shanghai suburb: A rural community survey. J Alzheimers Dis. (2014) 39:883–9. doi: 10.3233/JAD-131601, PMID: 24321893

[25] Katzman R Zhang MY Ouang-Ya-Qu Wang ZY Liu WT Yu E. A Chinese version of the Mini-Mental State Examination; Impact of illiteracy in a Shanghai dementia survey. J Clin Epidemiol. (1988) 41:971–8. doi: 10.1016/0895-4356(88)90034-0, PMID: 3193141

[26] Calvin CM Conroy MC Moore SF Kuzma E Littlejohns TJ. Association of multimorbidity, disease clusters, and modification by genetic factors with risk of dementia. JAMA Netw Open. (2022) 5(6):e2232124. doi: 10.1001/jamanetworkopen.2022.32124, PMID: 36125811 PMC9490497

[27] Grande G Marengoni A Vetrano DL Roso-Llorach A Rizzuto D Zucchelli A. Multimorbidity burden and dementia risk in older adults: The role of inflammation and genetics. Alzheimers Dement. (2021) 17:768–76. doi: 10.1002/alz.12237, PMID: 33403740 PMC8247430

[28] Song YH Niu JP Ji Y Zhang YW Peng RQ Li MD. Prevalence and risk factors of dementia in elder adults in Xiamen, China: A cross-sectional study. Psychogeriatrics. (2023) 23:71–6. doi: 10.1111/psyg.12904, PMID: 36353810

[29] Kuzuya M. Era of geriatric medical challenges: Multimorbidity among older patients. Geriatr Gerontol Int. (2019) 19:699–704. doi: 10.1111/ggi.13742, PMID: 31397060

[30] Jia LF Du YF Chu L Zhang ZJ Li FY Lyu DY. Prevalence, risk factors, and management of dementia and mild cognitive impairment in adults aged 60 years or older in China: A cross-sectional study. Lancet Public Health. (2020) 5:E661–71. doi: 10.1016/S2468-2667(20)30185-7, PMID: 33271079

[31] Mecocci P Boccardi V. The impact of aging in dementia: It is time to refocus attention on the main risk factor of dementia. Ageing Res Rev. (2021) 65:101210. doi: 10.1016/j.arr.2020.101210, PMID: 33186671

[32] Fayosse A Nguyen DP Dugravot A Dumurgier J Tabak AC Kivimäki M. Risk prediction models for dementia: Role of age and cardiometabolic risk factors. BMC Med. (2020) 18(1):107. doi: 10.1186/s12916-020-01578-x, PMID: 32423410 PMC7236124

[33] Zheng B Su BW Price G Tzoulaki I Ahmadi-Abhari S Middleton L. Glycemic control, diabetic complications, and risk of dementia in patients with diabetes: Results from a large UK cohort study. Diabetes Care. (2021) 44:1556–63. doi: 10.2337/dc20-2850, PMID: 34035076

[34] Koch M Jensen MK. HDL-cholesterol and apolipoproteins in relation to dementia. Curr Opin Lipidol. (2016) 27:76–87. doi: 10.1097/MOL.0000000000000257, PMID: 26709470 PMC4703432

[35] Chiu HY Chang HT Chan PC Chiu PY. Cholesterol levels, hormone replacement therapy, and incident dementia among older adult women. Nutrients. (2023) 15(20):4481. doi: 10.3390/nu15204481, PMID: 37892556 PMC10610485

[36] Veronese N Koyanagi A Dominguez LJ Maggi S Soysal P Bolzetta F. Multimorbidity increases the risk of dementia: A 15 year follow-up of the SHARE study. Age Ageing. (2023) 52(4):afad052. doi: 10.1093/ageing/afad052, PMID: 37078753 PMC10116948

[37] Xu ZJ Zhang DX Sit R Wong C Tiu J Chan D. Incidence of and risk factors for mild cognitive impairment in chinese older adults with multimorbidity in hong kong. Sci Rep. (2020) 10(1):4137. doi: 10.1038/s41598-020-60901-x, PMID: 32139719 PMC7057945

[38] Hu HY Zhang YR Aerqin Q Ou YN Wang ZT Cheng W. Association between multimorbidity status and incident dementia: A prospective cohort study of 245,483 participants. Transl Psychiatry. (2022) 12(1):505. doi: 10.1038/s41398-022-02268-3, PMID: 36476644 PMC9729184

[39] Kuo SC Lai SW Hung HC Muo CH Hung SC Liu LL. Association between comorbidities and dementia in diabetes mellitus patients: Population-based retrospective cohort study. J Diabetes Complications. (2015) 29:1071–6. doi: 10.1016/j.jdiacomp.2015.06.010, PMID: 26233574

[40] Leelakanok N D’Cunha RR. Association between polypharmacy and dementia - a systematic review and metaanalysis. Aging Ment Health. (2019) 23:932–41. doi: 10.1080/13607863.2018.1468411, PMID: 29746153

[41] Kristensen RU Norgaard A Jensen-Dahm C Gasse C Wimberley T Waldemar G. Changes in the prevalence of polypharmacy in people with and without dementia from 2000 to 2014: A nationwide study. J Alzheimers Dis. (2019) 67:949–60. doi: 10.3233/JAD-180427, PMID: 30689562

[42] Chen H Zhou YG Huang LY Xu XL Yuan CZ. Multimorbidity burden and developmental trajectory in relation to later-life dementia: A prospective study. Alzheimers Dement. (2023) 19:2024–33. doi: 10.1002/alz.12840, PMID: 36427050

